# Underlying hemodynamic differences are associated with responses to tilt testing

**DOI:** 10.1038/s41598-021-97503-0

**Published:** 2021-09-09

**Authors:** Artur Fedorowski, Giulia Rivasi, Parisa Torabi, Madeleine Johansson, Martina Rafanelli, Irene Marozzi, Alice Ceccofiglio, Niccolò Casini, Viktor Hamrefors, Andrea Ungar, Brian Olshansky, Richard Sutton, Michele Brignole, Gianfranco Parati

**Affiliations:** 1 Department of Clinical Sciences, Lund University, Malmö, Sweden; 2 Department of Cardiology, Skåne University Hospital, Carl-Bertil Laurells gata 9, 214 28 Malmö, Sweden; 3 Syncope Unit, Division of Geriatrics and Intensive Care Unit, Careggi Hospital, University of Florence, Florence, Italy; 4 Division of Cardiology, Department of Internal Medicine, University of Iowa Hospitals, Iowa City, IA USA; 5 Department of Cardiology, National Heart and Lung Institute, Imperial College, Hammersmith Hospital Campus, London, UK; 6 Faint & Fall Programme, IRCCS Istituto Auxologico Italiano, Ospedale San Luca, Milano, Italy; 7 Arrhythmology Centre and Syncope Unit, Department of Cardiology, Ospedali del Tigullio, Lavagna, Italy; 8 Department of Medicine and Surgery, University of Milano Bicocca, Milan, Italy

**Keywords:** Cardiovascular diseases, Arrhythmias, Hypertension

## Abstract

Aim of this study was to explore whether differences in resting hemodynamic parameters may be associated with tilt test results in unexplained syncope. We analyzed age, gender, systolic (SBP), diastolic blood pressure (DBP) and heart rate (HR) by merging three large databases of patients considered likely to be of vasovagal reflex etiology, comparing patients who had tilt-induced reflex response with those who did not. Tilt-induced reflex response was defined as spontaneous symptom reproduction with characteristic hypotension and bradycardia. Relationship of demographics and baseline supine BP to tilt-test were assessed using logistic regression models. Individual records of 5236 patients (45% males; mean age: 60 ± 22 years; 32% prescribed antihypertensive therapy) were analyzed. Tilt-positive (n = 3129, 60%) vs tilt-negative patients had lower SBP (127.2 ± 17.9 vs 129.7 ± 18.0 mmHg, p < 0.001), DBP (76.2 ± 11.5 vs 77.7 ± 11.7 mmHg, p < 0.001) and HR (68.0 ± 11.5 vs 70.5 ± 12.5 bpm, p < 0.001). In multivariable analyses, tilt-test positivity was independently associated with younger age (Odds ratio (OR) per 10 years:1.04; 95% confidence interval (CI), 1.01–1.07, p = 0.014), SBP ≤ 128 mmHg (OR:1.27; 95%CI, 1.11–1.44, p < 0.001), HR ≤ 69 bpm (OR:1.32; 95%CI, 1.17–1.50, p < 0.001), and absence of hypertension (OR:1.58; 95%CI, 1.38–1.81, p < 0.001). In conclusion, among patients with suspected reflex syncope, younger age, lower blood pressure and lower heart rate are associated with positive tilt-test result.

## Introduction

Tilt-testing (TT) was first introduced for clinical diagnosis of reflex syncope in 1986^1^, and has over the past decades been criticized for failing to provide discrimination between reflex (vasovagal) and other more serious causes of syncope, such as arrhythmic syncope, despite its acceptable sensitivity and specificity in true positive and negative subjects^2^. Thus, it is claimed that TT offers limited diagnostic value in those for whom it is most needed^3^. In this study we aimed to offer an alternative explanation for why some subjects with likely reflex syncope faint during tilt testing and others do not. The ISSUE 3 (International study of syncope of unknown etiology) set out to identify patients with recurrent syncope, unexplained but thought likely to be of reflex origin in older patients who had received a dual-chamber rate-drop response pacemaker after documentation of an asystolic reflex episode^4^. Unexpectedly, patients with positive pre-implant-tilt were relatively unprotected against recurrent syncope during the pacing phase. In contrast, patients with negative pre-implant tilt-testing had a very low incidence of recurrent syncope^5^. The new interpretation of tilt-testing incorporated a susceptibility to vertical posture stress, possibly co-existing with any cause of syncope. This so-called ‘hypotensive susceptibility’ is caused by a vertically induced fluid shift, and is associated with the hypotensive (vasodepressor) aspect of reflex syncope^3^.

Recently, we have identified a specific cardiovascular profile in patients with reflex syncope, characterized by lower systolic blood pressure (SBP), elevated heart rate (HR), and diastolic (DBP) compared with the general population^6^. Other recent studies have emphasized the primary role of hemodynamic changes in the mechanism of impending reflex syncope during TT^7,8^. In this large 3 center series of consecutive suspected reflex syncope, we aimed to explore whether differences in resting hemodynamic parameters may be associated with tilt test results in patients with suspected reflex syncope.

## Methods

### Study population and design

We analyzed individual values of resting supine BP and HR collected between 2003 and 2019 in three large databases of patients who had undergone TT for unexplained syncope, in tertiary syncope investigation units of hospitals in Lavagna, Italy (n = 2798), Florence, Italy (n = 805), and Malmö, Sweden (n = 1633). Consecutive patients who had undergone TT for diagnosis of syncope were included. Those with classical orthostatic hypotension (OH) and suspected postural orthostatic tachycardia syndrome (POTS) were excluded. Hypertension was defined as a clinical diagnosis of hypertension and antihypertensive medication reported by the patient. Despite the long study period, indications, methodology and interpretation of TT results remained unchanged during this time and were very similar to the recommendations of the current ESC syncope guidelines^9^.

TT was performed according to the “Italian protocol” and endorsed by the most recent ESC guidelines on Syncope^9^. The protocol consisted of at least 10-min supine period, a 20-min passive phase at a tilt angle of 70°, followed by a 15-min nitroglycerine-potentiated phase (300–400 mcg administered sublingually) if syncope was not induced during the passive phase^10^. Positive response was defined as reproduction of spontaneous symptoms in the presence of bradycardia and hypotension^9^. In all three centers, baseline hemodynamic data were obtained in the supine position prior to TT, using validated non-invasive beat-to-beat hemodynamic monitors, Task Force Monitor (CNSystems Medizintechnik GmbH, Graz, Austria) in Florence and Lavagna, and Nexfin (BMEYE, Amsterdam, Netherlands) or Finapres Nova monitors (Finapres Medical Systems, PH Enschede, Netherlands) in Malmö^11,12^. The monitors were calibrated before measurement using brachial cuff and oscillometric method according to the manufacturer’s instructions. As the monitors render beat-to-beat data, an average value of a hemodynamically stable period of 10 (Florence and Lavagna) or 30 s (Malmö) was recorded in the database. Data on baseline heart rate were unavailable in one of the three study-centers (Florence, Italy). The patient information was de-identified before merging databases. Ethical approval for original data collection was obtained by an appropriate institutional review board: "Comitato Etico Regionale" of Regione Liguria, Italy for Lavagna cohort, "Comitato Etico Area Vasta Centro, AOU Careggi, Florence”, for Florence cohort, and Regional Ethical Review Board in Lund, Sweden for Malmö cohort, and all participants gave their written informed consent. According to Italian and Swedish law, the use of anonymous patient data previously collected for patient care, as was the case here, did not require additional ethical approval.

### Statistical analyses

We retrieved the following data: age, gender, resting SBP and DBP, HR, and use of antihypertensive drugs although data on HR were not available in the Florence dataset. All the datasets were subsequently merged into one study population. Additionally, rate-pressure product (HR x SBP), a marker of cardiovascular fitness and myocardial oxygen consumption^13^, was calculated for merged Lavagna and Malmö databases.

Continuous data are shown as mean ± standard deviation, whereas frequencies are used to describe categorical data. The method of Kolmogorov and Smirnov was used to check the normality of distributions. Continuous variables were compared by means of the paired Student’s t-test. Paired and multiple proportions were compared by means of Pearson’s chi-square test. Stepwise multiple regression analysis was used to identify the independent factors associated with TT positivity. Multivariable logistic regression was adjusted for age, gender, SBP, and HR, whereas presence of hypertension was adjusted for age, gender and HR only. In addition, the results of the three centers were separately analyzed.

Analyses were performed using the Statistical Analysis System Software (version 9.4; SAS Institute, Cary, NC, USA; https://www.sas.com/sv_se/home.html) and IBM SPSS Statistics software (version 26.0; SPSS Inc., Chicago, IL, USA; https://www.ibm.com/products/spss-statistics). Statistical significance was set at the 0.05 level and all p-values were two-sided.

## Results

### Study population

The total study population consisted of 5236 patients, 55% of whom were females, the mean age was 60 ± 22 years. A total of 1655 patients (32%) were prescribed antihypertensive therapy. TT induced reflex syncope in 3129 (60%) patients and did not induce reflex syncope in 2,107 (40%) patients. Five-hundred-and sixty tests were positive during passive TT, and 2569 during drug-potentiated phase. TT was positive in 61% of males and 59% of females.

### Comparison between patients with and without tilt-induced reflex response

**Figure 1:**
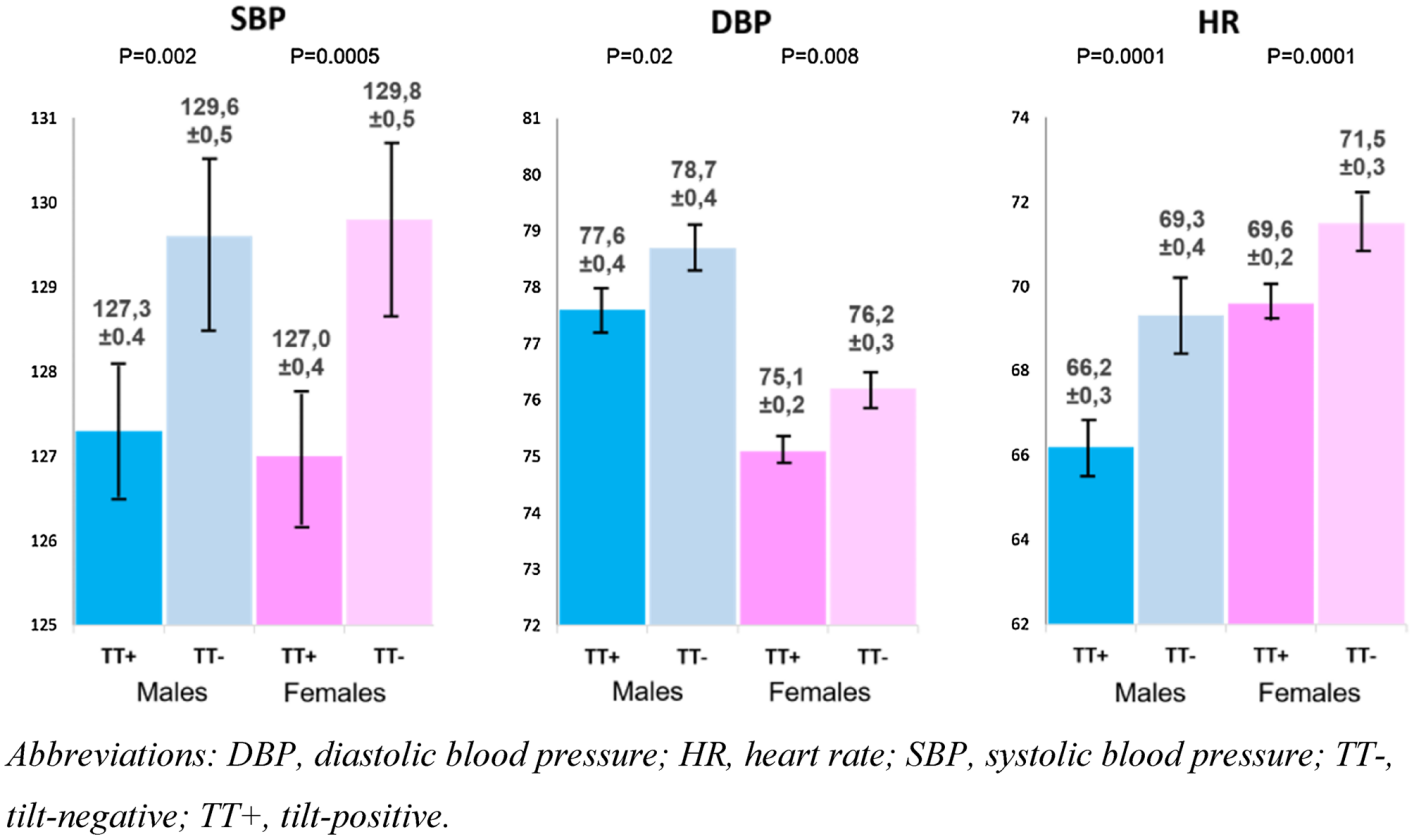
Gender-stratified systolic blood pressure, diastolic blood pressure and heart rate in tilt-positive and tilt-negative patients. *DBP *diastolic blood pressure;* HR *heart rate; *SBP* systolic blood pressure;* TT−* tilt-negative; *TT*+ tilt-positive. Resting systolic blood pressure, diastolic blood pressure and heart rate among tilt-positive and tilt-negative patients stratified by gender who were investigated using Italian tilt-test protocol for suspected syncope. Males, tilt-positive, n = 1428, tilt-negative, n = 923; females, tilt-positive, n = 1701, tilt-negative, n = 2107. All the values are shown as mean ± 1 SE. The bars show the ± 95% confidence limit (2 SEs).

Compared with TT-negative patients, TT-positive patients had lower baseline SBP (127.2 ± 17.9 vs 129.7 ± 18.4, p < 0.001), DBP (76.2 ± 11.5 vs 77.3 ± 11.7, p < 0.001) and HR (68.0 ± 11.5 vs 70.5 ± 12.5, p < 0.001). These differences were present both in males and females with small, but statistically significant, differences: baseline SBP was similar in males and females, but males had higher DBP whereas females had higher HR than males (p = 0.001 for DBP and HR) (Fig. 1).

### Comparison between subgroups

In univariate analyses (Table 1), we found a significantly higher rate of TT-induced reflex response among syncope patients below age 30 years, and in patients without history of hypertension. Lower baseline SBP below the mean value of 128 mmHg and lower baseline HR below the mean value of 69 bpm was also significantly associated with TT-induced reflex response. In a multivariable-adjusted logistic regression model (Table 1), younger age, lower BP and HR, and absence of hypertension remained the independent correlates of test positivity. In particular, a TT-induced reflex response was independently associated with younger age (Odds Ratio (OR) [per 10 years]: 1.04; 95% confidence interval (CI), 1.01–1.07, p = 0.007), SBP below 128 mmHg (OR: 1.27; 95%CI 1.11–1.44, p < 0.001), and HR below 69 bpm (OR:1.32; 95%CI 1.17–1.50, p < 0.001). Absence of hypertension diagnosis increased the probability of a positive TT-induced reflex response by over 50% (OR: 1.58; 95%CI 1.38–1.81, p < 0.001).

**Table 1:** Univariable and multivariable analysis of factors predicting tilt test positivity in patients investigated for unexplained syncope using the Italian tilt test protocol.

Variables	Total syncope population (n = 5236) N	Tilt-positive patients (n = 3129) n (%)	Univariable analysis p value	Multivariable analysis p value
**Age subgroups**				
10–29 years	748	493 (66)	0.006	0.016
30–59 years	1415	848 (60)		
≥ 60 years	3073	1788 (58)		
**Gender**				
Males	2351	1428 (61)	0.20	0.10
Females	2885	1701 (59)		
**Baseline SBP at the time of tilt testing**				
> 128 mmHg	2443	1358 (56)	0.0001	0.0001
≤ 128 mmHg	2793	1771 (63)		
**Baseline HR at the time of tilt testing**				
> 69 bpm	2088	1176 (56)	0.0001	0.0001
≤ 69 bpm	2324	1490 (64)		
Hypertension*				
Yes	1655	864 (52)	0.0001	0.0001
No	3581	2265 (63)		

*SBP *systolic blood pressure*, HR *heart rate*.*

*Adjusted for age, gender and heart rate.

Exclusion of one database did not substantially affect the overall results except for the impact of low SBP on TT positivity, which was attenuated after exclusion of Lavagna data in the multivariable-adjusted (but not univariate) logistic regression model (p = 0.083). Tilt testing results and resting hemodynamic parameters stratified according to the study center are shown in Table S1. The associations of BP with tilt positivity were weaker in Florence cohort, being significant for DBP only. The rate-pressure product was significantly lower among TT-positive patients across age and sex strata with exception of youngest females < 30 years (Figure S1). All rate-pressure product values were within the normal range (< 12,000)^13^.

## Discussion

In this three-center analysis of syncope patients with high pre-test probability of reflex mechanism, we found a slightly different hemodynamic pattern among those who were tilt-test positive. Males and females who had induction of reflex syncope during TT had lower baseline SBP, DBP, and HR compared with those who did not. We propose that patients more resistant to orthostatic stress induced by TT, who have slightly higher BP and HR, may have greater hemodynamic reserve in the face of gravitational challenge.

### Possible interpretation of tilt test results

Our results suggest that patients with TT-induced reflex syncope may have increased resting vagal tone and/or lower sympathetic tone, both expressed by lower HR, and narrower hemodynamic margins in the face of orthostatic stress, expressed by lower initial BP. Hypotensive susceptibility is suspected to exist in most people, particularly in individuals with a history of syncope^3^. Its identification is thought to play a key role in the management of syncope and selection of effective therapy, such as guiding pacemaker therapy in reflex syncope^14^, as well as to be the reason for the good response to pacing in tilt-negative patients^5^. In writing of the European Society of Cardiology Guidelines on Syncope 2018, the concept of hypotensive susceptibility was further extended to anticipate that some patients with reflex syncope were constitutionally predisposed to reflex syncope based on presenting a low blood pressure—‘a low blood pressure phenotype’^9^.

We have previously reported that individuals investigated due to unexplained syncope of likely reflex mechanism have in general lower SBP, but higher HR and DBP compared with normal population^6^. According to this study, patients who reproduce reflex syncope during TT have lower not only SBP, but also DBP and HR, and may thus represent a more vulnerable subset of syncope population. Recent studies have indicated that TT positivity is associated with neuroendocrine activation characterized by excess epinephrine and vasopressin release^15,16^, whereas adrenomedullin seems to play a protective role, probably reducing vessel permeability and acting against intravascular volume reduction during orthostasis^15^. Further, older age and higher resting SBP have been suggested to predict lower susceptibility to positive tilt-testing^15,17^. These findings were supported by Lindenberger et al. who observed upregulation of vasopressin release, reduced cardiac filling and cardiac output in women prone to reflex syncope^18^. It might be interpreted that vasopressin upregulation is a secondary effect of a relative hypovolemia, compared with non-vasovagal subjects, suggesting a key role of discrete water and electrolyte imbalance in increased tendency to syncope. There, also, remains a tenable possibility that the documented endocrine changes are precipitated by the developing adverse hemodynamic picture i.e. reduced venous return and cardiac filling. Combination of lower circulating blood volume and excessive blood pooling in capacitance vessels during orthostatic challenge may abruptly reduce stroke volume during TT, with a compensatory HR increase, as shown by Buszko et al.^19^.

Those with a more pronounced TT sensitivity demonstrate increased epinephrine and vasopressin release during tilt testing and thus appear to be in greater need of circulatory compensation as their resting SBP and DBP are lower^15,17^. This may provoke response from the central nervous system, leading to reflex syncope in extreme situations^20,21^. As previously shown, this response occurs when cerebral tissue oxygenation is strongly compromised^22^.

Although female gender, especially at younger age, is associated with higher syncope incidence^23^, only younger age but not gender was independently predictive of TT positivity. The protective factors against TT intolerance were resting SBP above 128 mmHg, heart rate above 69 bpm and presence of hypertension, even while prescribed antihypertensive treatment.

Higher HR might indicate less vagal tone and more efficient chronotropic compensatory mechanisms. As SBP mainly increases with greater stroke volume, reduced arterial elastance, and, to a lesser degree, with elevated peripheral vascular resistance^24^, higher SBP may suggest that those who are capable of tolerating TT have greater intravascular volume, better cardiac filling, and higher systemic vascular resistance compared with those who developed vasovagal syncope during TT. Interestingly, the association of combined heart rate and systolic blood pressure with TT positivity was markedly attenuated in younger women, who are genetically most predisposed to reflex syncope^25^, a fact that deserves more studies. Hypertension, on the other hand, implies chronic sympathetic activation with increased total peripheral resistance and elevated arterial tone in the precapillary vascular bed^26^. Arterial hypertension is detrimental for long-term cardiovascular integrity and promotes end-organ damage^27^, but hypertensive patients seem to be more resistant to orthostatic stress, either due to altered hemodynamic reserve, increased circulating blood volume, chronic neuroendocrine activation and arterial vasoconstriction or by baroreceptor resetting.

The differences in observed hemodynamic parameters between tilt-positive and tilt-negative patients were small, yet highly statistically significant. Despite these differences having limited clinical applications, they are important for clarifying pathophysiological mechanisms behind reflex syncope susceptibility in some individuals. Future studies might broaden the concept of hemodynamic prerequisite for reflex syncope by using Modelflow estimates of cardiac stroke volume and by applying prolonged and more accurate BP and HR assessment methodology such as 24-h ambulatory BP monitoring and heart-rate variability, as well as peripheral vascular resistance, blood volume, and venous return estimation.

### Strengths and limitations

The strengths of this three-center study lie in the large number of patients included and almost identical examination protocol, thus minimizing potential inter-center variability. There are some limitations that should be mentioned. Data on baseline heart rate were unavailable in one of the study centers (Florence, Italy), however, despite this limitation, the results in the remaining two study-centers demonstrated consistency. The study is retrospective with all typical limitations for retrospective analyses. Lower overall stress and sympathetic drive could have contributed to the lower SBP, DBP and HR observed in patients with TT-induced reflex syncope. We are unable to confirm or refute this possibility, however, this does not contradict our study hypothesis of a hemodynamic phenotype contributing to reflex syncope susceptibility. Further, patients without induction of reflex syncope during TT may have suffered syncope due to competing mechanisms such as cardiac arrhythmia but the final diagnoses in this patient group were not available. Finally, in believing that the constitutional nature of a low blood pressure phenotype contributes importantly to hypotensive susceptibility, we found it necessary to include a wide range of ages and hypertensive patients, some of whom were likely to have a similar susceptibility prior to developing their hypertensive disease. Our clinical observations suggest also that some patients reporting a history of reflex syncope in youth that resolves in mid-life, sustain recurrence of syncope once effective hypotensive therapy is introduced and hypertension treated^9,28^. Thus, the inclusion of hypertensive patients was deliberate in order to pursue the population concept as hypertension is very common in the general population, although we accept that this inclusion constitutes a limitation.

## Conclusions

Patients who develop reflex syncope during tilt testing have significantly lower blood pressure and heart rate compared with tilt-negative patients. In contrast, advanced age and hypertension are two important factors diminishing tilt-testing positivity. Our findings support the concept of a low heart rate and low blood pressure phenotype contributing to reflex syncope susceptibility during orthostatic stress.

## Supplementary Information

Supplementary Information.
