# Supplementary material for: Underlying hemodynamic differences are associated with responses to tilt testing
Source: Sci Rep. 2021 Sep 9;11:17894. doi: 10.1038/s41598-021-97503-0 (PMC8429732; doi:10.1038/s41598-021-97503-0)
Supplement: Supplementary file 1 — Supplementary Information. [file 41598_2021_97503_MOESM1_ESM.pdf]

Supplementary Material

Underlying hemodynamic differences are associated with responses to tilt testing

Artur Fedorowski[1,2*] MD, PhD, Giulia Rivasi[3] MD, Parisa Torabi[1] MD, Madeleine Johansson[1,2] MD, PhD, Martina Rafanelli[3] MD, Irene Marozzi[3] MD, Alice Ceccofiglio[3] MD, Niccolò Casini[3] MD, Viktor Hamrefors[1] MD, PhD, Andrea Ungar[3] MD, PhD, Brian Olshansky[4], MD, Richard Sutton [2,5] MB BS, DSc, Michele Brignole[6,7] MD, and Gianfranco Parati [6,8] MD

Affiliations:

1. Department of Clinical Sciences, Lund University, Malmö, Sweden,
2. Department of Cardiology, Skåne University Hospital, Malmö, Sweden
3. Syncope Unit, Division of Geriatrics and Intensive Care Unit, University of Florence and Careggi Hospital, Florence, Italy
4. Department of Internal Medicine, Division of Cardiology, University of Iowa Hospitals, Iowa City, IA, USA
5. Dept. of Cardiology, National Heart and Lung Institute, Imperial College, Hammersmith Hospital Campus, London, UK
6. IRCCS Istituto Auxologico Italiano, Faint & Fall programme, Ospedale San Luca, Milano, Italy
7. Arrhythmology Centre and Syncope Unit, Dept of Cardiology, Ospedali del Tigullio, Lavagna, Italy
8. Department of Medicine and Surgery, University of Milano Bicocca, Milan, Italy

Supplementary Tables

Table S1. Tilt testing results and resting hemodynamic parameters. Analyses stratified according to the study center.

	**Lavagna**			**Florence**			**Malmö**		
	**TT+ n=1,632**	**TT- n=1,166**	**P-value**	**TT+ n=450**	**TT- n=355**	**P-value**	**TT+ n=1,047**	**TT- n=586**	**P-value**
**SBP, mmHg ± SD**	124.2±15.7	127.0±16.1	<0.001	130.5±17.2	132.0±18.0	0.23	130.4±20.3	133.9±21.8	<0.001
**DBP, mmHg ± SD**	76.8±11.3	78.3±11.5	<0.001	81.7±12.2	79.9±11.4	0.03	73.0±10.4	73.8±11.6	0.15
**HR, bpm ± SD**	67.4±11.7	69.5±12.3	<0.001	N/A			69.0±11.2	72.5±12.8	<0.001
**Hypertension, n, (%)**	416 (25%)	379 (32%)	<0.001	240 (53%)	210 (59%)	0.10	208 (20%)	202 (34%)	<0.001

Abbreviations: Bpm, beats per minute; DBP, diastolic blood pressure; HR, heart rate; SBP, systolic blood pressure; SD, standard deviation; TT-, tilt-negative; TT+, tilt-positive.

Supplementary Figures

Figure S1. Age- and gender-stratified rate-pressure product (heart rate x systolic blood pressure) in tilt-positive and tilt-negative patients of Lavagna and Malmo cohorts (n=4,431).

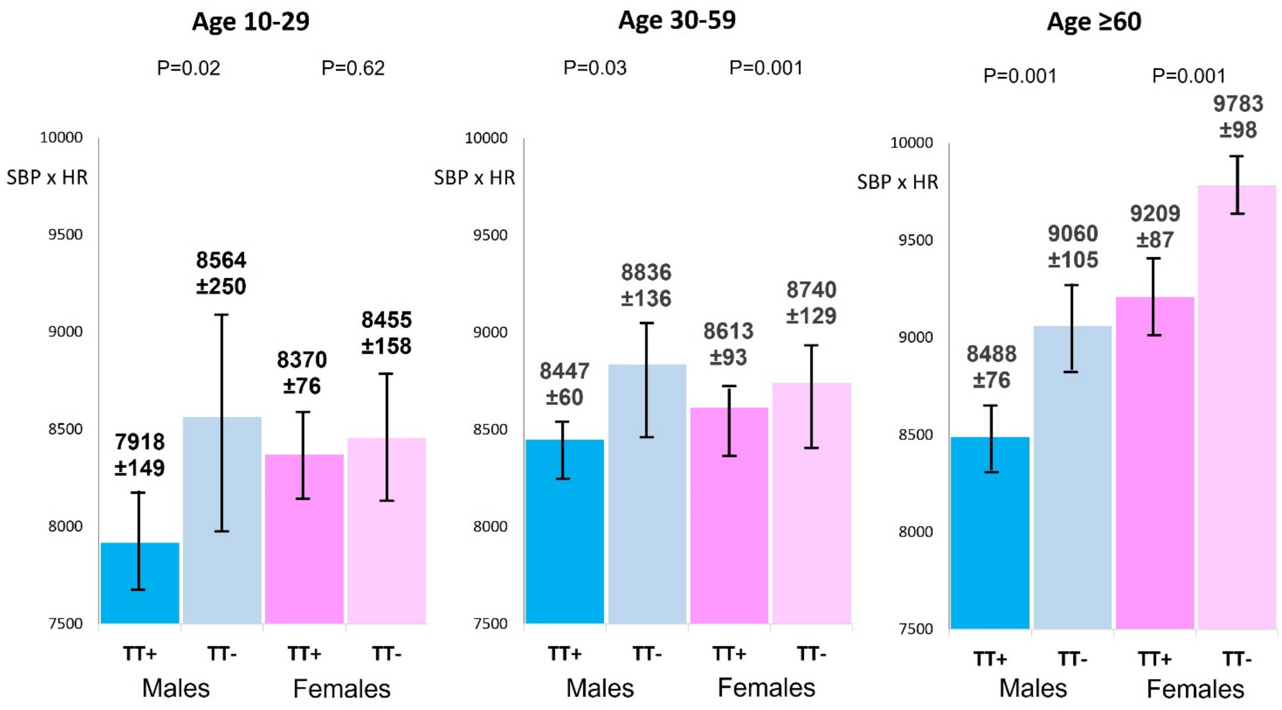

Abbreviations: HR, heart rate; SBP, systolic blood pressure; TT-, tilt-negative; TT+, tilt-positive. All the values are shown as mean ± 1 SE. The bars show the ±95% confidence limit (2 SEs)
